# Genetic risk factors associated with gestational diabetes in a multi-ethnic population

**DOI:** 10.1371/journal.pone.0261137

**Published:** 2021-12-20

**Authors:** Paula Benny, Hyeong Jun Ahn, Janet Burlingame, Men-Jean Lee, Corrie Miller, John Chen, Johann Urschitz

**Affiliations:** 1 Department of Obstetrics, Gynecology, and Women’s Health, John A. Burns School of Medicine, University of Hawaii, Honolulu, HI, United States of America; 2 Department of Quantitative Health Sciences, John A. Burns School of Medicine, University of Hawaii, Honolulu, HI, United States of America; 3 Department of Anatomy, Biochemistry and Physiology, John A. Burns School of Medicine, University of Hawaii, Honolulu, HI, United States of America; PLOS, UNITED KINGDOM

## Abstract

**Aims:**

Genome-wide association studies have shown an increased risk of type-2-diabetes (T2DM) in patients who carry single nucleotide polymorphisms in several genes. We investigated whether the same gene loci confer a risk for gestational diabetes mellitus (GDM) in women from Hawaii, and in particular, Pacific Islander and Filipino populations.

**Methods:**

Blood was collected from 291 women with GDM and 734 matched non-diabetic controls (Pacific Islanders: 71 GDM, 197 non-diabetic controls; Filipinos: 162 GDM, 395 controls; Japanese: 58 GDM, 142 controls). Maternal DNA was used to genotype and show allele frequencies of 25 different SNPs mapped to 18 different loci.

**Results:**

After adjusting for age, BMI, parity and gravidity by multivariable logistic regression, several SNPs showed significant associations with GDM and were ethnicity specific. In particular, SNPs rs1113132 (EXT2), rs1111875 (HHEX), rs2237892 (KCNQ1), rs2237895 (KCNQ1), rs10830963 (MTNR1B) and rs13266634 (SLC30A8) showed significant associations with GDM in Filipinos. For Japanese, SNPs rs4402960 (IGFBP2) and rs2237892 (KCNQ1) were significantly associated with GDM. For Pacific Islanders, SNPs rs10830963 (MTNR1B) and rs13266634 (SLC30A8) showed significant associations with GDM. Individually, none of the SNPs showed a consistent association with GDM across all three investigated ethnicities.

**Conclusion:**

Several SNPs associated with T2DM are found to confer increased risk for GDM in a multiethnic cohort in Hawaii.

## Introduction

Normal pregnancy is associated with a period of relative insulin resistance that progressively leads to maternal hyperglycemia and transplacental passage of glucose to promote fetal growth. This is similar to the type of insulin resistance found in patients with type 2 diabetes (T2DM) [1–5]. Maternal hyperglycemia usually results in an increase in insulin secretion by maternal pancreatic beta-cells [6]. However, in approximately 1 out of 15 pregnant women in the USA [7], the diagnosis of gestational diabetes (GDM) is made when this compensatory mechanism is impaired. It has been postulated that there may be an underlying baseline level of insulin resistance that predates the pregnancy and that changes of glycemic regulation associated with the secretion of human placental lactogen, also known as human chorionic somatomammotropin, from the placenta, leads to a compromise in glycemic control and the diagnosis of GDM.

Maternal age, overweight/obese status and a family history of diabetes are risk factors for GDM [8, 9]. Recent studies have also described combinatorial effects of risk factors in the manifestation of GDM, together with the integration of first trimester fasting glycemia measurements [10, 11]. Fetuses of pregnant women with poorly controlled GDM are at an increased risk for macrosomia, polyhydramnios, birth injury, neonatal hypoglycemia, and other neonatal metabolic disturbances. In addition to these perinatal complications, the likelihood of long-term consequences for the health and wellbeing of both mothers and their offspring is significantly higher compared to normoglycemic pregnancies. GDM is not only associated with a higher incidence of adult obesity and the development of T2DM later in life, but also with a predisposition for cardiovascular diseases [12, 13] and metabolic disorders [14–16]. While GDM usually resolves after delivery, the increased probability of future deterioration in insulin resistance and beta-cell insufficiency puts women who have had GDM at a greater risk of developing T2DM than those who had a normal pregnancy [17]. Women with a previous history of GDM have a 35% to 60% chance of developing diabetes in the next 10–20 years [18].

GDM prevalence rates have been reported to vary in direct proportion to the prevalence of T2DM in several ethnic populations [8]. Along with high rates of T2DM, GDM is particularly prevalent in certain minority populations including Asian, Native American, Hispanic and African-American women, who are at higher risk for GDM than non-Hispanic Caucasian women [19]. The local prevalence of T2DM in Hawaii is increasing, especially among Asian and Pacific Islander parturients. These groups have demonstrated significantly higher rates of impaired glucose tolerance (15.5%) and T2DM (20.4%) compared to the overall U.S. population [20]. This increase in T2DM in Asian and Pacific Islanders is likely to correlate with increasing number of pregnancies complicated by GDM [20, 21]. A recent report on the prevalence of GDM in Hawaii found that GDM rates were highest in Filipino women, followed by Chinese, Japanese and Native Hawaiian/Pacific Islander women. Caucasians in Hawaii had the lowest GDM rate of all ethnic groups [22]. Such differences in GDM incidences are likely to be multi-factorial and are expected to include genetic factors [23].

Several pioneering genome-wide association studies (GWAS) have identified over 60 genetic loci in genes responsible for insulin secretion, insulin resistance, lipid and glucose metabolism, and other pathways that are associated with type 2 diabetes [24–29]. While most of these studies were from European populations, more recent GWAS have identified novel loci specific to other populations such as South Asians [30], Japanese [31, 32], Mexicans [33], Chinese [34, 35], African Americans [36, 37], and Asian Indians [38]. Since women with GDM are at increased risk of T2DM and both T2DM and GDM are polygenic, multifactorial diseases, it has been suggested that both diseases are manifestations of similar pathophysiological processes [39]. Several studies have validated these loci to also infer an increased risk of gestational diabetes [40–43]. While these studies have examined several different ethnic populations, few prior studies have investigated the specific associations of T2DM in the multiethnic Hawaiian populations [44]. One such study to focus on the Hawaiian population is the MEC-PAGE (Multi-Ethnic Cohort Population Architecture using Genomics and Epidemiology). While this large study provided data on risk assessments of chronic diseases such as diabetes [45], colorectal [46] and prostate cancer [47] in Native Hawaiians, this was predominantly in an older population (age 45–75 years old). Another recent study focused on disease occurrence in Native Hawaiians and identified a locus on Chr6 associated with T2DM [48]. However, these loci could not be validated as the replication study size was a small, predominantly Samoan cohort. As Native Hawaiians comprise approximately 10% of the State’s population, a more accurate representation of the different populations of Hawaii is currently lacking.

Therefore, in this study, we investigated whether a select panel of 25 significantly reported single nucleotide polymorphisms (SNPs) known to be associated with T2DM conferred a risk for GDM in Pacific Islander, Filipino and Japanese women in a unique cohort of pregnant women living in Hawaii. The results of this cross-sectional study would provide new knowledge on the genetic risks of GDM susceptibility occurring in the distinct populations of Hawaii.

## Materials and methods

### Subjects

A case–control study was performed comparing genotype frequencies of 25 different SNPs (Table 1) in healthy pregnant women and pregnant women with GDM. Ethical approval for the study was obtained from the Institutional Review Board (IRB) of the University of Hawaii. All participants provided written informed consent. Samples were obtained from the RMATRIX Biorepository of the John A. Burns School of Medicine, University of Hawaii following the study’s inclusion/exclusion criteria. The Hawaiian Biorepository houses over 9,000 biospecimens collected from pregnant women from 2006–2012. All pregnant women had been routinely screened with a 1-hour 50-g oral glucose challenge test (GCT) at 24–28 weeks of gestation. Gestational age was determined by ultrasound examination. Individuals with blood glucose levels greater than 130 mg/dL underwent a 3-hour 100-g oral glucose tolerance test (OGTT) in the morning after an overnight hour fast.

**Table 1:** Clinical characteristics of participants according to ethnic group.

Ethnicity	Variable	Control					GDM					*p*
		N	Mean	Std Dev	Min	Max	N	Mean	Std Dev	Min	Max	
**Filipino**	**Maternal Age**	395	30.5	5.7	18.0	44.0	162	31.7	5.5	19.0	42.0	**0.029**
	**BMI**	395	24.2	4.4	15.4	43.5	155	25.9	5.2	15.9	44.6	**<0.001**
	**Gravida**	395	2.8	1.7	1.0	13.0	162	2.8	1.6	1.0	8.0	0.930
	**Parity**	395	1.2	1.1	0.0	6.0	162	1.4	1.3	0.0	5.0	**0.031**
**Japanese**	**Maternal Age**	142	35.0	4.8	19.0	45.0	58	35.8	5.0	27.0	50.0	0.300
	**BMI**	141	22.4	3.6	15.7	43.5	51	24.9	3.8	17.7	34.5	**<0.001**
	**Gravida**	142	2.5	1.5	1.0	7.0	58	3.2	1.9	1.0	7.0	**0.020**
	**Parity**	142	0.9	0.9	0.0	4.0	58	0.9	1.1	0.0	4.0	0.950
**Pacific Islander**	**Maternal Age**	197	28.0	5.9	18.0	43.0	71	30.0	6.1	19.0	46.0	**0.016**
	**BMI**	192	29.4	6.2	16.5	50.0	56	32.7	7.1	18.3	47.8	**<0.001**
	**Gravida**	197	3.4	2.2	1.0	12.0	71	3.3	2.3	1.0	12.0	0.870
	**Parity**	197	2.0	1.9	0.0	8.0	71	1.8	1.8	0.0	8.0	0.410

Pregnant women were diagnosed with GDM when 2 or more glucose values were at or above the specified Coustan-Carpenter thresholds for diagnosing gestational diabetes; fasting 95 mg/dL, 1-hour 180 mg/dL, 2-hour 155 mg/dL, and 3-hour 140 mg/dL [49]. Blood was collected from 291 women who met Coustan-Carpenter criteria for GDM and 734 non-diabetic controls (normal glucose tolerance). Only subjects, where self-reported ethnicity was concordant with that of all four biological grandparents’ ethnicity were included in this study. According to the US Census Bureau, race and ethnicity are defined as a person’s self-identification with one or more social groups [50]. Subjects may report multiple races and ethnicities based on geographical, social, and cultural affiliations associated with human civilization. Self-identified population stratifications and geographic ancestry have been demonstrated to be highly correlated with self-identified race/ethnicity as well as a determinant of genetic structure [51]. The GDM cohort consisted of 71 Pacific Islander, 162 Filipino, and 58 Japanese women from Hawaii. The control cohort consisted of 197 Pacific Islander, 395 Filipino, and 142 Japanese women from Hawaii with normal glucose tolerance (NGT). Information regarding Height (m), weight (kg), age, BMI, ethnicity (self-reported) and GCT, OGTT results were obtained from prenatal records.

### Genotyping

The 25 single nucleotide polymorphisms (SNPs) were chosen due to their strong association with T2DM in other ethnic groups including Caucasians/United States [25], Europeans [26] and Caucasians/Canada [27]. While these SNPs were associated with T2DM in other ethnic groups, this is the first study to evaluate the association in Native Hawaiian, Pacific Islander, Filipino and Japanese populations. Genomic DNA was extracted from whole blood using the Autopure DNA isolation system (Gentra Systems, Minneapolis, MN) following the manufacturer’s protocol. All SNPs selected for genotyping (Table 2) were candidate or GWAS-identified variants shown to be associated with T2DM or GDM. Genotyping was performed on the TaqMan OpenArray platform (Life Technologies, Foster City, CA) using 125ng of genomic DNA. TaqMan OpenArray Genotyping Mastermix was added to the DNA and this solution was then distributed across the OpenArray plates pre-loaded with TaqMan genotyping assays for 32 SNPs. Polymerase chain reaction (PCR) was performed on a Bio-Rad Slide Chambers Dual-Block Alpha unit using PCR conditions set by the manufacturer. Allelic discrimination was done on a Biotrove OpenArray System using the OpenArray SNP Genotyping Analysis Software Version 1.0.3. All SNPs were analyzed using TaqMan Genotyper Software Version 1.0.1. Quality control criteria included fingerprint blanks, which are used to identify the plate and verify cross-contamination and or sample error and no template controls (NTC), which serve as a background detector and review of any reagent problems. DNA samples that failed in more than 25% of genotyping assays were excluded, assuming poor DNA quality and uncertain measurements. A genotyping error of 0.3% was determined by blind duplicate genotyping. Automated call rates for all 25 SNPs exceeded 97%.

**Table 2:** Association of 25 SNPs with GDM risk.

		Filipino			Japanese			Pacific Islanders		
Gene	SNP	Alleles	MAF	HWE	Alleles	MAF	HWE	Alleles	MAF	HWE
ADCY5	rs2877716	**C**/T	0.7	0.028	**C**/T	0.3	1.000	**C**/T	4.3	1.000
ARAP 1	rs11603334	G/**A**	5.5	1.000	G/**A**	3.4	1.000	G/**A**	5.5	0.415
CDKAL1	rs7754840	G/**C**	49.6	0.258	G/**C**	39.4	0.718	G/**C**	45.6	0.877
CDKN2A/2B	rs10811661	**T**/C	30.8	0.285	**T**/C	42.4	0.300	**T**/C	36.4	0.754
DGKB	rs2191349	**T**/G	24.3	0.476	**T**/G	31.9	0.565	**T**/G	15.1	0.030
EXT2	rs1113132	**C**/G	27.1	0.082	G/C*	34.3	0.856	**C**/G	14.2	0.546
FTO	rs8050136	C/**A**	15.5	0.843	C/**A**	16	1.000	C/**A**	11.7	0.737
G6PC2	rs3755157	C/**T**	35.0	0.571	C/**T**	38.2	0.258	**T**/C*	48.5	0.748
G6PC2	rs16856187	A/**C**	37.3	0.330	A/**C**	33.9	0.573	A/**C**	43.8	1.000
G6PC2	rs560887	C/**T**	2.0	0.015	C/**T**	1.8	1.000	C/**T**	1.2	<0.010
GCK	rs4607517	G/**A**	13.9	<0.010	G/**A**	22.7	1.000	G/**A**	18.9	0.815
GCKR	rs780094	**C**/T	41.7	0.210	T/C*	49.2	0.735	**C**/T	27.2	0.207
HHEX	rs1111875	T/**C**	17.0	0.032	T/**C**	30.9	1.000	T/**C**	35.7	0.02
HHEX	rs7923837	A/**G**	9.5	0.325	A/**G**	18.7	0.567	A/**G**	27.1	0.077
IGFBP2	rs4402960	G/**T**	26.5	0.358	G/**T**	29.4	0.551	G/**T**	22.4	0.399
KCNQ1	rs2237892	**C**/T	38.7	0.462	**C**/T	40.9	0.601	**C**/T	28.7	1.000
KCNQ1	rs2237895	A/**C**	33.1	0.406	A/**C**	32.3	0.848	A/**C**	24.1	1.000
MADD	rs10501320	**G**/C	0.5	1.000	**G**/C	0.3	1.000	**G**/C	1.2	1.000
MTNR 1B	rs10830963	**G**/C	47.8	0.606	C/G*	43.2	0.286	**G**/C	48.9	0.281
PKN2	rs6698181	C/**T**	18.8	0.623	C/**T**	40.2	0.476	C/**T**	8.7	1.000
SLC30A8	rs13266634	T/**C**	49.9	0.749	**C**/T*	38.8	0.858	**C**/T*	41.9	0.084
TCF7L2	rs7903146	C/**T**	1.8	1.000	C/**T**	5.2	1.000	C/**T**	11.5	0.041
TCF7L2	rs7901695	T/**C**	1.7	1.000	T/**C**	5.2	1.000	T/**C**	9.7	0.399
TCF7L2	rs12255372	G/**T**	1.7	1.000	G/**T**	3.6	1.000	G/**T**	9.2	0.688
TCF7L2	rs11196205	G/**C**	4.1	0.134	G/**C**	6.7	1.000	G/**C**	11.2	0.720

Alleles: major/minor, MAF = Major Allele Frequency, HWE = Hardy-Weinburg Equilibrium. SNPs not at Hardy–Weinberg equilibrium (p<0.05) are shown in blue. Reversed minor allele compared to Filipino Controls are in red with an asterisk. Risk alleles are bold and underlined.

### Statistical analysis

Demographic and clinical variables (age, BMI, gravidity, and parity) were summarized by descriptive statistics; means, standard deviations, minimums and maximums. Differences in patient characteristics between case and control groups for each ethnicity were examined using independent two-sample t-test and differences in patient characteristics among the three race/ethnicity groups were also examined using one-way Analysis of Variance (ANOVA).

Genotype minor allele frequencies (MAF) were summarized by percentages. The Breslow-Day test [52] was used to assess the homogeneity of MAFs and odds ratios (OR) among the three ethnic groups. We used chi-square tests to determine whether an individual polymorphism was in Hardy–Weinberg equilibrium. For the dominant and recessive model of each polymorphism, unadjusted odds ratios were calculated with p values using chi-square tests within each ethnic group. The dominant/recessive model was chosen over other models such as the additive or multiplicative model as it increased the resolution of assessing disease risk, similar to other studies of diabetes [53, 54]. We evaluated the homogeneity of the unadjusted OR among the three ethnic groups by Breslow-Day test and used Mantel-Haenszel formula [55] to calculate summary unadjusted OR with 95% confidence interval (CI). We then selected SNPs which showed that 1) unadjusted OR were significantly different from 1.0; 2) p values of Breslow-Day test were less than 0.05; 3) the summary of unadjusted OR was significantly different from 1.0 based on Mantel-Haenszel formula. For the selected SNPs, multivariable logistic regression models were used to further adjust and control for confounding factors, such as age, BMI, parity, and gravidity. All data analyses were performed in R-package and SAS 9.3 and a two-tailed p<0.05 was regarded as statistically significant.

## Results

We compared the genotype frequencies in 291 women from Hawaii who met Coustan-Carpenter criteria for GDM and 734 matched non-diabetic controls (Pacific Islander: 71 subjects with GDM and 197 non-diabetic controls; Filipinos: 162 GDM patients and 395 controls; Japanese: 58 GDM patients and 142 controls).

The clinical baseline characteristics are listed in Table 1. The mean BMI was significantly higher in all three GDM groups (Filipino and Japanese p<0.001, Pacific Islander p = 0.016) when compared to the ethnically matched controls. Additionally, mean maternal age (p = 0.029) and mean parity (p = 0.031) were significantly higher in Filipino women with GDM than in their control (p = 0.001), whereas parity (p = 0.020) and maternal age differed in the Japanese GDM cohort and Pacific Islander women respectively.

25 SNPs reported to be associated with T2DM were genotyped. Table 2 reports the SNP alleles, as well as minor allele frequency (MAF) and the results from the Hardy–Weinberg equilibrium analysis in the control group for all 25 SNPs studied. All SNPs were in Hardy–Weinberg equilibrium at the significance level of p>0.05 except for (i) rs4607517 (GCK) and rs2877716 (ADCY5) in Filipinos, (ii) rs2191349 (DGKB) and rs7903146 (TCF7L2) in Pacific Islanders, (iii) rs1111875 (HHEX) and rs560887 (G6PC2) in both Filipino and Pacific Islanders (Table 2, blue values). For certain SNPs, there was also a reversal of major/minor alleles among the subpopulations (as in an allele that is major in one race becomes minor in another race and vice versa) (Table 2, red alleles).

Unadjusted odds ratios (OR) and associated p-values were subsequently calculated for the association of genotypes with GDM in the dominant and recessive model of each polymorphism (Tables 3 and 4 respectively). Interestingly, for each race, several SNPs showed significant associations with GDM (Tables 3 and 4, blue values). However, none of the SNPs appeared to show a consistent association with GDM across all three investigated ethnicities. To evaluate this observation further, we assessed the homogeneity of the unadjusted OR among the three ethnic groups using the Breslow-Day (BD) test as well as the Mantel-Haenszel (MH) formula that calculates a summary of unadjusted OR with 95% confidence interval. In the dominant model, the association of rs2237892 (KCNQ1, Table 3) with GDM differed significantly between our populations (BD p = 0.024, green value, Table 3). Indeed, while highly significant for Filipinos (p<0.001), we detected only a moderate association of rs2237892 (KCNQ1) with GDM in Japanese subjects (p = 0.03) and none in Pacific Islanders (p = 0.49) (Table 3). Similarly, for the recessive model, rs13266634 (SLC30A8, Table 4) showed a significant association with GDM in Pacific Islanders (p = 0.005), but not in Filipinos (p = 0.301) or Japanese (p = 0.230) (BD p = 0.004, green value, Table 4). SNPs with values from Mantel-Haenszel formula that were significantly different from 1.0 in the dominant and recessive models are highlighted in red in Tables 3 and 4, respectively.

**Table 3:** Association of SNPs with GDM risk using a dominant model (unadjusted odds ratios; OR, Breslow-Day test; BD, Mantel-Haenszel formula; MH).

		Filipino				Japanese				PI							
Gene	SNP	OR		P		OR		P		OR		P		BD_FJPI	OR.mh	Low. B	Up. B
**ADCY5**	**rs2877716**	0.39	(	0.337	)	0.61	(	0.176	)	0.27	(	**0.046**	)	0.889	0.34	0.11	1.04
ARAP 1	rs11603334	1.11	(	0.725	)	1.08	(	0.906	)	0.76	(	0.601	)	0.821	1.02	0.64	1.63
CDKAL1	rs7754840	1.5	(	0.071	)	0.66	(	0.195	)	1.82	(	0.089	)	0.057	1.27	0.92	1.75
CDKN2A/2B	rs10811661	0.88	(	0.499	)	1.17	(	0.641	)	0.57	(	0.054	)	0.248	0.83	0.63	1.11
DGKB	rs2191349	1.13	(	0.507	)	0.78	(	0.433	)	0.74	(	0.365	)	0.406	0.96	0.72	1.29
**EXT2**	**rs1113132**	1.52	(	**0.028**	)	4.00	(	**0.032**	)	1.04	(	0.907	)	0.218	**1.48**	**1.09**	**2.02**
FTO	rs8050136	1.11	(	0.604	)	0.9	(	0.767	)	0.7	(	0.335	)	0.539	0.97	0.71	1.34
G6PC2	rs16856187	0.74	(	0.12	)	0.68	(	0.217	)	1.17	(	0.626	)	0.393	0.80	0.60	1.07
**G6PC2**	**rs3755157**	0.83	(	0.346	)	0.52	(	**0.047**	)	1.18	(	0.676	)	0.252	0.80	0.59	1.07
G6PC2	rs560887	0.44	(	0.156	)	0.4	(	0.35	)	1.43	(	0.293	)	0.745	0.41	0.15	1.12
GCK	rs4607517	1.45	(	0.088	)	1.17	(	0.632	)	0.91	(	0.753	)	0.456	1.22	0.90	1.65
GCKR	rs780094	0.89	(	0.56	)	1.41	(	0.367	)	0.67	(	0.17	)	0.307	0.89	0.66	1.19
**HHEX**	**rs1111875**	1.5	(	**0.047**	)	0.61	(	0.13	)	1.43	(	0.229	)	0.053	1.22	0.91	1.63
**HHEX**	**rs7923837**	1.51	(	0.085	)	1.19	(	0.605	)	1.84	(	**0.036**	)	0.607	**1.52**	**1.11**	**2.08**
**IGFBP2**	**rs4402960**	0.97	(	0.888	)	0.5	(	**0.031**	)	0.83	(	0.534	)	0.205	0.82	0.62	1.09
**KCNQ1**	**rs2237892**	0.49	(	**< .001**	)	0.5	(	**0.033**	)	1.22	(	0.488	)	**0.024**	** **		
**KCNQ1**	**rs2237895**	1.1	(	0.624	)	0.96	(	0.907	)	1.79	(	**0.047**	)	0.28	1.20	0.91	1.59
MADD	rs10501320	0.59	(	0.627	)	0.61	(	0.176	)	1.43	(	0.293	)	0.556	0.43	0.10	1.95
**MTNR 1B**	**rs10830963**	0.63	(	**0.025**	)	0.71	(	0.368	)	0.52	(	**0.037**	)	0.799	**0.61**	**0.45**	**0.83**
PKN2	rs6698181	0.89	(	0.551	)	0.82	(	0.555	)	1.02	(	0.95	)	0.91	0.89	0.66	1.22
**SLC30A8**	**rs13266634**	0.58	(	**0.011**	)	0.74	(	0.387	)	1.09	(	0.781	)	0.239	**0.72**	**0.53**	**0.98**
TCF7L2	rs11196205	0.95	(	0.89	)	0.7	(	0.465	)	0.82	(	0.583	)	0.878	0.84	0.54	1.31
TCF7L2	rs12255372	0.92	(	0.876	)	0.64	(	0.496	)	0.72	(	0.411	)	0.902	0.76	0.43	1.34
TCF7L2	rs7901695	0.91	(	0.866	)	0.8	(	0.683	)	0.92	(	0.817	)	0.979	0.89	0.52	1.50
TCF7L2	rs7903146	1.2	(	0.724	)	0.8	(	0.683	)	0.67	(	0.288	)	0.655	0.82	0.48	1.38

BD-FJPI = Breslow Day Test for Filipino, Japanese and Pacific Islander. SNPs with significant unadjusted OR p values (p<0.05) are highlighted in blue. SNPs with significant Breslow-Day test p values (p<0.05) are highlighted in green. SNPs with values from Mantel-Haenszel formula significantly different from 1.0 are highlighted in red.

**Table 4:** Association of SNPs with GDM risk using a recessive model (unadjusted odds ratios; OR, Breslow-Day Test; BD, Mantel-Haenszel formula; MH).

		Filipino				Japanese				PI							
Gene	SNP	OR		P		OR		P		OR		P		BD_FJPI	OR. mh	Low. B	Up. B
ADCY5	rs2877716	1.13	(	0.99	)	NA	(	NA	)	NA	(	NA	)	NA	0	0	0
ARAP 1	rs11603334	2.39	(	0.544	)	NA	(	NA	)	2.85	(	0.471	)	0.931	2.605	0.364	18.643
CDKAL1	rs7754840	0.82	(	0.375	)	1.38	(	0.441	)	1.33	(	0.436	)	0.366	0.997	0.71	1.4
**CDKN2A/2B**	**rs10811661**	0.63	(	0.167	)	0.36	(	**0.031**	)	1.17	(	0.701	)	0.18	0.667	0.425	1.047
DGKB	rs2191349	1.48	(	0.327	)	0.94	(	0.903	)	0.31	(	0.204	)	0.32	1.057	0.587	1.903
EXT2	rs1113132	0.73	(	0.392	)	1.52	(	0.201	)	1.14	(	0.332	)	0.131	1.03	0.652	1.627
FTO	rs8050136	1.06	(	0.923	)	2.56	(	0.267	)	1.95	(	0.484	)	0.662	1.514	0.655	3.499
G6PC2	rs3755157	1.04	(	0.898	)	1.49	(	0.374	)	1.19	(	0.621	)	0.792	1.168	0.788	1.732
G6PC2	rs560887	1.13	(	0.99	)	NA	(	NA	)	1.14	(	1	)	0.372	0.518	0.06	4.461
G6PC2	rs16856187	1.04	(	0.898	)	1.44	(	0.406	)	1.36	(	0.387	)	0.745	1.198	0.821	1.749
GCK	rs4607517	1.02	(	0.969	)	1.06	(	0.935	)	0.48	(	0.46	)	0.794	0.919	0.438	1.929
GCKR	rs780094	0.76	(	0.268	)	1.10	(	0.785	)	1.35	(	0.6	)	0.521	0.9	0.616	1.316
HHEX	rs1111875	1.13	(	0.798	)	1.1	(	0.857	)	1.15	(	0.729	)	0.998	1.128	0.679	1.873
HHEX	rs7923837	1.81	(	0.448	)	1.65	(	0.595	)	1.3	(	0.592	)	0.923	1.456	0.703	3.013
IGFBP2	rs4402960	0.75	(	0.435	)	0.37	(	0.156	)	0.84	(	0.826	)	0.679	0.665	0.361	1.224
**KCNQ1**	**rs2237892**	0.55	(	**0.03**	)	0.78	(	0.565	)	0.82	(	0.736	)	0.726	0.634	0.412	0.977
**KCNQ1**	**rs2237895**	2	(	**0.015**	)	0.69	(	0.517	)	1.64	(	0.404	)	0.25	**1.604**	**1.025**	**2.508**
MADD	rs10501320	NA	(	NA	)	NA	(	NA	)	NA	(	NA	)	NA	0	0	0
**MTNR 1B**	**rs10830963**	0.39	(	**< .001**	)	0.61	(	0.15	)	0.7	(	0.296	)	0.339	**0.506**	**0.356**	**0.72**
PKN2	rs6698181	0.59	(	0.325	)	1.89	(	0.117	)	1.14	(	1	)	0.185	1.2	0.653	2.205
**SLC30A8**	**rs13266634**	0.79	(	0.301	)	0.55	(	0.23	)	2.77	(	**0.005**	)	**0.004**	** **		
TCF7L2	rs11196205	1.13	(	0.99	)	NA	(	NA	)	1.14	(	0.57	)	0.363	0.432	0.052	3.622
TCF7L2	rs12255372	NA	(	NA	)	NA	(	NA	)	1.14	(	1	)	NA	0	0	0
**TCF7L2**	**rs7903146**	1.13	(	0.296	)	NA	(	NA	)	1.14	(	0.345	)	**0.028**	** **		
TCF7L2	rs7901695	NA	(	NA	)	NA	(	NA	)	1.14	(	0.571	)	NA	0	0	0

BD-FJPI = Breslow Day Test for Filipino, Japanese and Pacific Islander. SNPs with significant unadjusted OR p values (p<0.05) are shown in blue. SNPs with significant Breslow-Day test p values (p<0.05) are shown in green. SNPs with values from Mantel-Haenszel formula significantly different from 1.0 are shown in red.

Next, we used multivariable logistic regression models to adjust for age, BMI, gravidity, and parity. Such regression analyses were applied only to selected SNPs that originally showed either significant unadjusted OR p values (p<0.05; blue) or significant Breslow-Day test p values (p<0.05; green) or values from Mantel-Haenszel formula significantly different from 1.0 (red) in the dominant and recessive models shown in Tables 3 and 4. The results of the regression analyses for the SNPs that originally showed significant unadjusted OR p values (p<0.05) (blue values in Tables 3 and 4) are shown in Table 5. The results of the regression analyses for the SNPs that originally showed significant Breslow-Day test p values (p<0.05) (green values in Tables 3 and 4) are shown in Table 6. The results of the regression analyses for the SNPs that showed values from Mantel-Haenszel formula significantly different from 1.0 (red values in Tables 3 and 4) are shown in Table 7.

**Table 5:** Multivariable logistic regression analysis (OR).

			Filipino				Japanese				PI			
	Gene	SNP	OR		P_value		OR		P_value		OR		P_value	
**Dominant**	ADCY5	rs2877716									0.37	(	0.153	)
	**EXT2**	rs1113132	1.53	(	**0.034**	)	7.40	(	0.063	)				
	G6PC2	rs3755157					0.57	(	0.133	)				
	**HHEX**	rs1111875	1.60	(	**0.027**	)								
	HHEX	rs7923837									1.55	(	0.196	)
	**IGFBP2**	rs4402960					0.32	(	**0.003**	)				
	**KCNQ1**	rs2237892	0.5	(	**< .001**	)	0.45	(	**0.035**	)				
	KCNQ1	rs2237895									1.72	(	0.108	)
	**MTNR 1B**	rs10830963	0.62	(	**0.028**	)					0.42	(	**0.021**	)
	**SLC30A8**	rs13266634	0.49	(	**0.002**	)								
**Recessive**	**KCNQ1**	rs2237892	0.51	(	**0.019**	)								
	**KCNQ1**	rs2237895	1.84	(	**0.046**	)								
	CDKN2A/2B	rs10811661					0.35	(	0.053	)				
	**MTNR 1B**	rs10830963	0.34	(	**< .001**	)								
	**SLC30A8**	rs13266634									2.43	(	**0.03**	)

List of SNPs (adjusted for age, BMI, parity, gravidity) which showed significant unadjusted OR p values (p<0.05) in the Dominant (Table 3) and Recessive (Table 4).

**Table 6:** Multivariable logistic regression analysis (BD).

			Filipino				Japanese				PI			
	Gene	SNP	OR		P_value		OR		P_value		OR		P_value	
**Dominant**	**KCNQ1**	rs2237892	0.5	(	**< .001**	)	0.45	(	**0.035**	)	1.18	(	0.622	)
**Recessive**	TCF7L2	rs7903146	NA	(	0.99	)	NA	(	NA	)	NA	(	0.98	)
	**SLC30A8**	rs13266634	0.73	(	0.17	)	0.62	(	0.41	)	2.43	(	**0.03**	)

List of SNPs (adjusted for age, BMI, parity, gravidity) which showed significant Breslow-Day test p values (p<0.05) in the Dominant (Table 3) and Recessive models (Table 4).

**Table 7:** Multivariable logistic regression analysis.

			Filipino				Japanese				PI			
	Gene	SNP	OR		P_value		OR		P_value		OR		P_value	
**Dominant**	EXT2	rs1113132	1.33	(	0.13	)	6.05	(	0.085	)	1.16	(	0.69	)
	HHEX	rs7923837	1.48	(	0.095	)	1.01	(	0.97	)	1.56	(	0.17	)
	**MTNR 1B**	rs10830963	0.63	(	**0.025**	)	0.81	(	0.59	)	0.39	(	**0.006**	)
	**SLC30A8**	rs13266634	0.56	(	**0.006**	)	0.73	(	0.38	)	1.02	(	0.96	)
**Recessive**	KCNQ1	rs2237895	1.5	(	0.14	)	0.61	(	0.46	)	1.19	(	0.8	)
	**MTNR 1B**	rs10830963	0.37	(	**< .001**	)	0.52	(	0.092	)	0.56	(	0.14	)

List of SNPs (adjusted for age, BMI, parity, gravidity) which showed values from Mantel-Haenszel formula significantly different from 1.0 in the Dominant (Table 3) and Recessive (Table 4) models.

Overall, after adjusting for age, BMI, parity and gravidity, SNPs rs1113132 (EXT2), rs1111875 (HHEX), rs2237892 (KCNQ1), rs10830963 (MTNR1B) and rs13266634 (SLC30A8) showed significant associations with GDM in the dominant model for Filipinos, while rs2237892 (KCNQ1), rs2237895 (KCNQ1) and rs10830963 (MTNR1B) were significantly associated with GDM in the recessive model (Table 8). For Japanese women, the SNPs rs4402960 (IGFBP2) and rs2237892 (KCNQ1) showed significant associations with GDM in the dominant model, but no SNP was significantly associated with GDM in the recessive model (Table 8). For Pacific Islander women, the SNP rs10830963 (MTNR1B) showed significant association with GDM in the dominant model, while rs13266634 (SLC30A8) was significantly associated with GDM in the recessive model (Table 8).

**Table 8:** Summary table illustrating SNPs with significant associations with GDM, according to race.

			Filipino				Japanese				PI			
	Gene	SNP	OR		P_value		OR		P_value		OR		P_value	
**Dominant**	**EXT2**(*)	rs1113132	1.53	(	**0.034**	)								
	**HHEX**(*)	rs1111875	1.60	(	**0.027**	)								
	**IGFBP2**(*)	rs4402960					0.32	(	**0.003**	)				
	**KCNQ1**(*)(**)	rs2237892	0.5	(	**< .001**	)	0.45	(	**0.035**	)				
	**MTNR 1B** (*)(***)	rs10830963	0.62	(	**0.028**	)					0.42	(	**0.021**	)
	**SLC30A8**(*)(***)	rs13266634	0.56	(	**0.006**	)								
**Recessive**	**KCNQ1**(*)	rs2237892	0.51	(	**0.019**	)								
	**KCNQ1**(*)	rs2237895	1.84	(	**0.046**	)								
	**MTNR 1B**(*)(***)	rs10830963	0.37	(	**< .001**	)								
	**SLC30A8**(*)(**)	rs13266634									2.43	(	**0.03**	)

The table lists SNPs that showed significant unadjusted OR p values (p<0.05; *), significant Breslow-Day test p values (p<0.05; **) or values from Mantel-Haenszel formula significantly different from 1.0 (***) in the Dominant (Table 3) and Recessive (Table 4) models. Significance was determined after multivariable logistic regression analysis was performed to control for confounding factors.

## Discussion

GDM and T2DM are both multifactorial diseases and are believed to share similar epidemiologic risk factors such as obesity, hypertension, polycystic ovarian syndrome, racial-ethnic background, and family history. Several studies have demonstrated a shared genetic susceptibility for both T2DM and GDM among various populations. Cho *et al*. investigated various SNPs known to be associated with an increased risk of T2DM in women with and without GDM in a Korean population [40]. Allelic differences in CDKAL1 (Cdk5 regulatory associated protein 1-like 1), CDKN2A-2B (cyclin-dependent kinase inhibitor 2A), HHEX (hematopoietically expressed homeobox), IGFT2BP2 (Insulin Like Growth Factor Binding Protein 2), SLC30A8 (Solute Carrier Family 30 Member 8), and TCF7L2 (Transcription Factor 7 Like 2) conferred an increased risk of GDM [40]. More recently, MTNR1B (rs10830962) was also found to have an excess association with T2DM and GDM in a Korean population [56]. rs2237895 on the KCNQ1 gene has been confirmed to be associated with both GDM and T2DM in Pakistani and Chinese cohorts [57, 58]. Gene variants of HMG20A (rs7178572) and HNF4A (rs4812829) showed significant association with GDM in Asian Indians [59].

The unique multiethnic population of Hawaii is comprised of both immigrants and multigenerational descendants of Japanese, Chinese, and Filipino heritage, in addition to those of Native Hawaiian ancestry. While many patients presenting for care identify as multiethnic, the inclusion criteria for this study population was strictly limited to those self-reporting one ethnicity according to all 4 grandparents. This allowed us to compare across multiple ethnicities at once without disproportionate heterogeneity. Identifying genotypic differences may account for the variation in disease phenotypes that are seen in various populations of pregnant women. For example, an East Asian cohort of women living in New York City with GDM were found to have a normal BMI, have a low risk for fetal macrosomia and GDM phenotype that was relatively easy to control with only diet modification [60]. However, a cohort of Arabian women with GDM living in Scandinavia were found to be much more insulin-resistant than Caucasian Scandinavian pregnant women diagnosed with GDM using a homeostasis model assessment [61]. Of note, multiple testing adjustments such as Bonferroni or Benjamini-Hochberg corrections were not included in this study owing to the modest sample size. This was to maintain the capacity to discover any potential associations between the selected SNPs, ethnicity and diabetes in this unique population.

Looking at allelic variations in our population demonstrates the importance of identifying phenotypic factors to screen for gestational diabetes as well as investigating drug treatments that target ethnic-specific mechanisms that can be used to develop personalized medicine. For instance, KCNQ1 gene encodes for potassium inwardly rectifying channel subfamily J, member 11 [62], and polymorphisms associated with potassium channels are likely to control insulin secretion in the pancreatic islet cells. The dominant model demonstrated SNPs at the CDKN2A loci, a gene coding for a cyclin-dependent kinase inhibitor 2A, were highly associated with an increased risk of GDM in Japanese participants. The results from our candidate gene approach are also consistent with prior studies showing B-cell dysfunction and impaired insulin secretion in Japanese populations [63]. Upstream of this locus are sequences that encode for p15INK4b and p16 INK4a, which both inhibit CDK4 and decrease pancreatic beta cell replication [64].

MTNR1B polymorphisms also have associations with GDM in Filipino and Pacific Islander populations. rs10830963 has been reported to have a strong correlation in a meta-analysis of GDM GWAS studies [65], and this finding has been seen among Korean, Greek [66], Russian [67] and Chinese women [42]. MTNR1A and MTNR1B encode for melatonin receptors and high-risk allele carriers at rs10830963 also have higher expression of MTNR1B in pancreatic beta cells, which leads to impaired insulin secretion [68]. Interestingly, the presence of this SNP was associated with a lower frequency of GDM in Pacific Islander women in our cohort. We observe that no single T2DM associated SNP is replicated across all Hawaiian populations who develop GDM, and this could be attributed to our modest sample size. However, we have refined the resolution of SNPs associated specifically with Japanese, Filipino and Pacific Islander populations in Hawaii. Future studies could validate these observations in a larger cohort. In addition, further studies could build on our findings and investigate a larger panel of candidate genes such as BACE2 and HKDC1 which are associated with maternal metabolic traits but were not included in this study [69].

## Conclusion

These findings elucidate the pathophysiology of increased risk of T2DM later in life for women affected by GDM and highlight the importance of lifestyle modifications to decrease this risk. Such associations need to be validated in larger Asian and Pacific Islander cohorts and further characterized into clinically meaningful phenotypes that can be designed to guide personalized medicine. While deleterious genetic mutations may provide increased susceptibility for gestational diabetes and impaired glucose tolerance postnatally, the environmental risk factors for this condition should also be considered for treatment.
